# Potential Mosquito Vectors for Shuni Virus, South Africa, 2014–2018

**DOI:** 10.3201/eid2712.203426

**Published:** 2021-12

**Authors:** Milehna Mara Guarido, Thopisang Motlou, Megan A. Riddin, Caitlin MacIntyre, Sontaga Cris Manyana, Todd Johnson, Maarten Schrama, Erin E. Gorsich, Basil D. Brooke, A. Paulo G. Almeida, Marietjie Venter

**Affiliations:** University of Pretoria, Pretoria, South Africa (M.M. Guarido, T. Motlou, M.A. Riddin, C. MacIntyre, S.C. Manyana, T. Johnson, A.P.G. Almeida, M. Venter);; Copperbelt University, Kitwe, Zambia (T. Johnson);; Leiden University, Leiden, the Netherlands (M. Schrama);; University of Warwick, Coventry, UK (E.E. Gorsich);; National Institute for Communicable Diseases/NHLS, Johannesburg, South Africa (B.D. Brooke);; University of the Witwatersrand, Johannesburg (B.D. Brooke);; NOVA University of Lisbon, Lisbon, Portugal (A.P.G. Almeida)

**Keywords:** Shuni virus, *Orthobunyavirus*, mosquitoes, disease vector, vector-borne diseases, viruses, South Africa

## Abstract

Shuni virus is associated with neurologic and febrile illness in animals and humans. To determine potential vectors, we collected mosquitoes in South Africa and detected the virus in species of the genera *Mansonia*, *Culex*, *Aedes*, and *Anopheles*. These mosquitoes may be associated with Shuni virus outbreaks in Africa and emergence in other regions.

The genus *Orthobunyavirus* (family *Peribunyaviridae*) includes emerging arthropodborne viruses associated with human and animal disease worldwide (*1*). In 1966, orthobunyavirus Shuni virus (SHUV) was isolated from a cow, *Culicoides* midges*,* and a febrile child in Nigeria (*2*); SHUV recently emerged in Israel, where it has been associated with birth defects in ruminants (*3*). SHUV has been associated with neurologic disease in horses and wildlife (*4*,*5*) and was recently implicated in human cases of neurologic disease in South Africa (*6*). SHUV was detected in field-caught *Culex theileri* mosquitoes in the 1970s (*5*), and *Culicoides* midges have been suggested as vectors (*7*). We investigated mosquitoes collected in northeastern parts of South Africa to identify their potential as vectors of orthobunyaviruses in the Simbu serogroup of arboviruses, including SHUV.

## The Study

**Figure 1:**
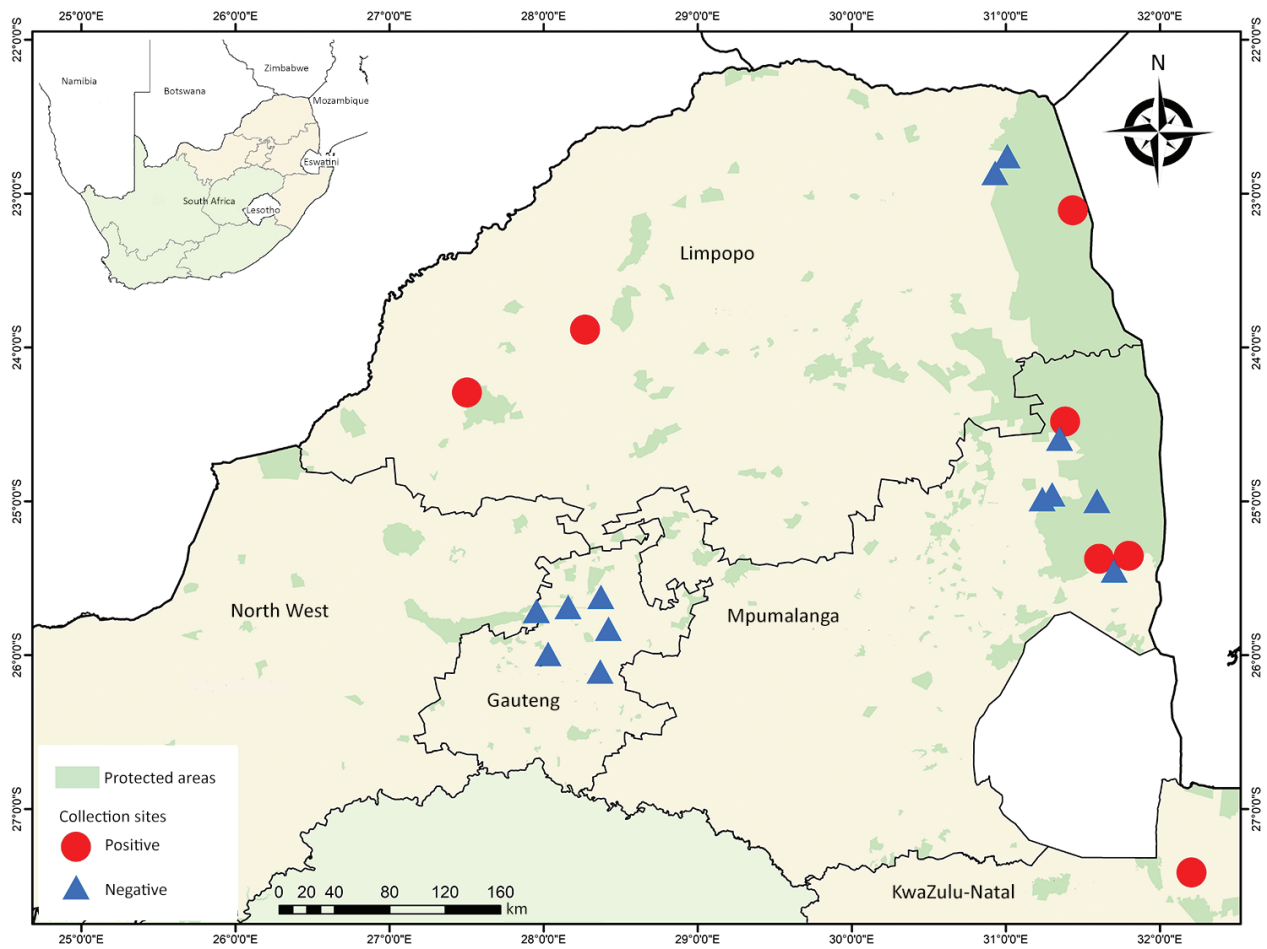
Mosquito collection sites indicating collection locations of Shuni virus–positive (circles) and negative (triangles) mosquito pools, South Africa, January 2014–May 2018. Inset map shows location of South Africa in Africa.

We collected mosquitoes across 5 provinces of South Africa (Figure 1). Site selection was based on historical outbreaks of arboviruses, including SHUV, in animals (*4*,*5*) and humans (*6*). During January 2014–May 2017, we collected mosquitoes monthly; we performed additional collections in 2017 in and around the Kruger National Park (*8*). In 2018, we performed 1 collection per site during January–May.

We used multiple types of dry ice (carbon dioxide) baited traps: nets, CDC miniature light traps (https://www.johnwhock.com), and BG-Sentinel traps (https://www.bg-sentinel.com). We set traps during 3:30–6:00 pm and emptied them during 5:00–8:00 am. We killed mosquitoes by freezing and then morphologically identified them to the species level. We pooled females (<50 individuals) by species, collection site, and month. We selected mosquitoes for screening from pools collected during January–June, which represents late summer and autumn, when arbovirus infections in animals and humans in South Africa increase. We obtained climate data from the South African Weather Service (http://www.weathersa.co.za).

For the virus assays, we produced homogenate pools by placing 5 sterile glass beads in microcentrifuge tubes containing 2 mL of reconstituted minimum essential medium, which we then vigorously shook and clarified. The resulted supernatant was stored at −80°C. To extract viral RNA from 200 μL homogenate, we used an RNeasy mini kit (QIAGEN, https://www.qiagen.com) according to the manufacturer’s instructions. We screened extracted RNA by using 2 PCRs, each targeting the nucleocapsid (NP) gene on the small (S) segment: a Simbu serogroup/orthobunyavirus–specific one-step TaqMan real-time reverse transcription PCR targeting a 152-bp fragment (*4*) and an SHUV nested real-time RT-PCR targeting a 460-bp fragment (*9*). In an attempt to obtain larger fragments, we performed an SHUV conventional PCR with published primers (*10*).

For mosquito barcoding (species identification), we extracted DNA from 50 μL of the homogenate by using a QIAGEN DNeasy Blood & Tissue Kit according to the manufacturer’s instructions. The subunit I of the cytochrome oxidase gene was amplified by using universal primers (*11*).

All products of the expected size were sequenced by Sanger sequencing at the Forestry Agriculture Bioinformatics Institute, University of Pretoria (Pretoria, South Africa). We compared the resulting sequences by using BLAST (https://blast.ncbi.nlm.nih.gov/Blast.cgi) with sequences available from GenBank, including SHUV strains from South Africa, Nigeria, and Israel and other representative members of Simbu serogroup. For the cytochrome oxidase gene, we selected representative mosquito sequences from GenBank and BOLD (https://v3.boldsystems.org). We compiled multiple sequence alignments by using MAFFT (https://mafft.cbrc.jp/alignment/software), produced maximum-likelihood trees by using MEGA 7.0 (https://www.megasoftware.net), and calculated maximum-likelihood estimates of mosquito infection rates by using PooledInfRate (https://www.cdc.gov/ncidod/dvbid/westnile/software.htm).

Of the 64,603 adult mosquitoes collected as described (*8*,*12*), we tested 39,035 females. A total of 11 pools were positive for SHUV (Table 1). No other orthobunyaviruses were detected. Positive pools for SHUV were detected in conservation areas (6/11, 54.5%) and rural areas (5/11, 45.5%) (Figure 1). Populations of the SHUV-positive mosquito species peaked with the heavy rains and with the highest mean air temperatures (Appendix Figure 1), which promote establishment of breeding sites and favorable habitats for developing stages and subsequent population growth.

**Table 1:** Mosquito species positive for Shuni virus, South Africa, January 2014–May 2018

Species	No. assayed	No. pools positive/no. pools tested	Infection rate, % (95% CI)*
*Anopheles pharoensis*	27	1/4	39.0 (2.4–212.0)
*Culex theileri*	508	1/22	1.9 (0.1–9.0)
*Cx. annulioris*	120	1/7	6.7 (0.5–33.6)
*Mansonia africana*	340	3/13	8.7 (2.6–23.3)
*Ma. uniformis*	2,428	2/62	0.8 (0.1–2.7)
*Aedes subargenteus*	1	1/1	Not applicable†
*Ae. mcintoshi*	3,653	1/87	0.3 (0.0–1.3)
*Aedes* spp.	273	1/25	3.6 (0.2–17.2)
Total	7,350	11/221	

*Maximum-likelihood estimation: no. positive/no. mosquitoes assayed × 1,000. †When all pools tested were positive for Shuni virus, the likelihood methods failed.

**Figure 2:**
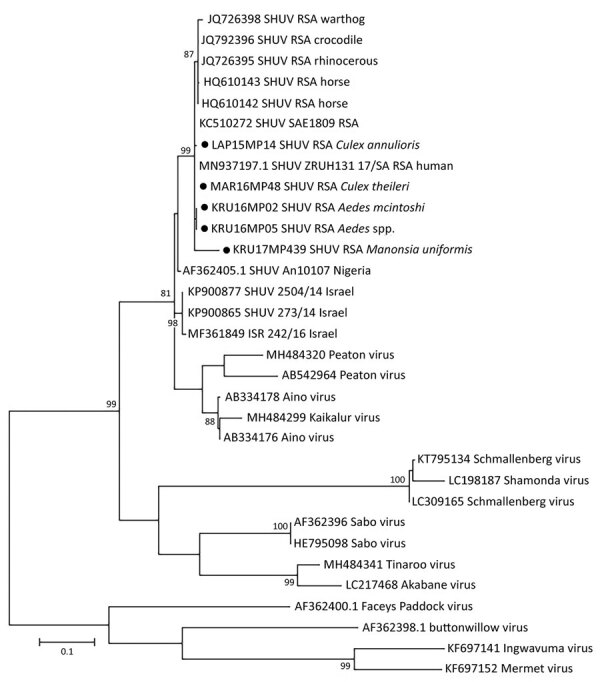
Phylogenetic tree of SHUV-positive homogenate mosquito pools, South Africa, January 2014–May 2018 (black dots), based on 32 sequences and 328 bp of the nucleocapsid gene on the small segment. The tree was constructed with MEGA 7 software (https://www.megasoftware.net) by using the maximum-likelihood method and the Kimura 2-parameter model with 1,000 bootstrap replicates and includes members of the Simbu serogroup. The tree with the highest log likelihood (−299.13) is shown. GenBank accession numbers are indicated for the new and reference strains, which were selected from SHUV strains identified in South Africa among horses and wildlife (*4*,*9*) as well as strains from Nigeria and Israel available in GenBank. Numbers on internal branches indicate bootstrap values. RSA, South Africa; SHUV, Shuni virus.

The maximum-likelihood phylogeny based on the genus *Orthobunyavirus* PCR fragment of 152-bp of the S segment showed that all SHUV viruses from the mosquitoes clustered with the Simbu serogroup (Appendix Figures 1, 2) and were closest to SHUV on the basis of p-distance analyses (data not shown). For 5 samples, a larger region of the S segment could be amplified to confirm the clustering with SHUV strains previously identified in horses and wildlife from South Africa (Figure 2) and p-distances of 94%–100% with strains previously identified in South Africa, Israel, and Nigeria. Mosquito barcodes consisting of 517-bp were used to build a maximum-likelihood tree (Appendix Table 1, Figures 1–3). The barcoding confirmed all morphologic identifications except for a pool of damaged *Aedes* spp. mosquitoes and for *Ae. subargenteus* mosquitoes (for which no other sequence was available in the databases).

Of the 11 pools of SHUV-positive mosquitoes, species belonged to the genera *Mansonia* (5 pools), *Aedes* (3 pools), *Culex* (2 pools), and *Anopheles* (1 pool) (Table 2). Previously, SHUV had been detected in *Cx. theileri* mosquitoes collected in the 1970s near Johannesburg, South Africa (*5*). In that study, 2 pools of SHUV-positive *Cx. theileri* mosquitoes were also identified, although mosquitoes of this species were not abundant in the sites detected.

**Table 2:** Mosquitoes positive for Shuni virus, South Africa, January 2014–May 2018*

Species	Site	ID no.	Pool size	GenBank SHUV accession no.	GenBank COI accession no.†
*Anopheles. pharoensis*	Marakele	Mar16mp59	12	NA	MT428079
*Culex theileri*	Marakele	Mar16mp48	3	MN914125	MT428080
*Cx. annulioris*	Lapalala	Lap15mp15	4	MN914124	MT428086
*Mansonia uniformis*	KNP	Knp17mp758	9	NA	MT428087
*Ma. africana*	KNP	Knp17mp761	41	NA	MT428089
*Ma. uniformis*	KNP	Knp17mp755	2	NA	MT428088
*Ma.* africana	KNP	Knp17mp753	1	NA	MT434140
*Aedes subargenteus*	Jozini	Kzn17mp108	2	NA	MT428082
*Ae. mcintoshi*	Mnisi	Kru16mp02	4	MT433095	MT428081
*Aedes* spp.	Mnisi	Kru16mp05	8	MT433096	MT428085
*Ma. uniformis*	Mnisi	Kru17mp439	50	MT433097	MT428083

*COI, subunit I of the cytochrome oxidase gene; ID, identification; KNP, Kruger National Park; NA, not available because of sequence <200 bp; SHUV, Shuni virus. †These sequences are available upon request.

The highest rate of SHUV detection was in *Mansonia uniformis* mosquitoes, which were found in high numbers at the Shuni virus–positive pool collection sites. Three other arboviruses have been isolated from *M. uniformis* mosquitoes in South Africa: Wesselsbron, Ndumu, and Spondeweni (*13*). *M. africana* mosquitoes tested positive, but only small numbers of these mosquitoes were collected. *Mansonia* spp. mosquitoes can feed readily on humans and animals (*13*) and could have a potential epidemiologic role as bridge species for transmission between animals and humans.

Mosquitoes of other species that tested positive included *Aedes mcintoshi* and *Ae. subargenteus.* Positive *Ae. mcintoshi* mosquitoes were collected from Mnisi, where they were the most abundant *Aedes* spp. at that site. They are considered nonspecific/opportunistic feeders and have a broad range of mammal hosts (*14*). *Ae. subargenteus* mosquitoes are tree hole mosquitoes and are either rare in South Africa (*14*) or are not attracted to the traps used in our study. Although little information about those mosquitoes is available, they might have a strong preference for biting humans (*14*).

Although SHUV has been detected in mosquitoes, recent studies have also implicated *Culicoides* spp. midges as potential competent vectors (*15*). An investigation of the vector competence of *Culicoides* midges and laboratory-reared *Cx. pipiens* and *Ae. aegypti* mosquitoes for SHUV (*7*) indicated that neither species of mosquito was susceptible but that *Culicoides* midges demonstrated the capacity to transmit SHUV. No *Ae. aegypti* and *Cx. pipiens* field-caught mosquitoes tested positive for SHUV in this or other studies. Vector competence studies that used SHUV-positive species of mosquitoes identified in our study may define appropriate mosquito vectors and their role in the transmission of SHUV to animals and humans in Africa and the risk to areas where they are found outside the continent.

## Conclusions

Entomologic surveillance for orthobunyaviruses revealed a wide range of potential mosquito vectors for SHUV. We identified SHUV in different species of mosquitoes in South Africa, where cases with neurologic signs have been detected in animals (*4*,*5*) and humans (*6*). The identified mosquito species have also been associated with other arboviruses across Africa. SHUV recently emerged in Israel, where it is associated with neurologic disease and birth defects in animals (*3*). Mosquitoes of the identified species are potential vectors of SHUV and may be associated with SHUV outbreaks in Africa and further emergence in new regions.

Appendix Supplemental results from study of potential mosquito vectors for Shuni virus, South Africa, 2014–2018.
