# Supplementary material for: Potential Mosquito Vectors for Shuni Virus, South Africa, 2014–2018
Source: Emerg Infect Dis. 2021 Dec;27(12):3142–6. doi: 10.3201/eid2712.203426 (PMC8632193; doi:10.3201/eid2712.203426)
Supplement: Appendix — Supplemental results from study of potential mosquito vectors for Shuni virus, South Africa, 2014–2018. [file 20-3426-Techapp-s1.pdf]

Article DOI: https://doi.org/10.3201/eid2712.203426

Potential Mosquito Vectors for Shuni Virus, South Africa, 2014–2018

Appendix

Appendix Table 1. Sequences that originated from GenBank or BOLD used at COI analyses and their origin.

Origin	Species	Accession no.
Kenya	*Aedes luteocephalus*	KU380354.1
Kenya	*Aedes luteocephalus*	KU380427.1
Ghana	*Aedes lilii*	LC507874.1
USA	*Aedes aegypti*	AF390098.2
Kenya	*Aedes aegypti*	KX536613.1
Uganda	*Aedes aegypti*	GQ165806.1
Liverpool strain	*Aedes aegypti*	AY056596.1
Kenya	*Aedes aegypti*	KX420482.1
Formosus strain	*Aedes aegypti*	AY056597.1
Kenya	*Aedes metallicus*	KU380468.1
Senegal	*Aedes metallicus*	MG242506.1
Kenya	*Aedes metallicus*	KU380376.1
Kenya	*Aedes metallicus*	KU187014.1
Kenya	*Aedes simpsoni*	KT881399.1
Kenya	*Aedes simpsoni*	KT881398.1
Kenya	*Aedes mcintoshi*	GBDCU1711–15
South Africa	*Aedes mcintoshi*	CULSA018–19
South Africa	*Aedes mcintoshi*	CULSA060–19
South Africa	*Aedes mcintoshi*	CULSA019–19
Kenya	*Aedes mcintoshi*	KJ940759.1
Kenya	*Aedes mcintoshi*	KU187018.1
Kenya	*Culex duttoni*	GBDP48191–19
Kenya	*Culex duttoni*	GBDP49051–19
South Africa	*Culex theileri*	GMSAT669–13
South Africa	*Culex theileri*	CULSA071–19
South Africa	*Culex theileri*	GMSAX726–14
Portugal	*Culex theileri*	GBDP49644–19
England	*Culex pipiens s.l.*	PIRCX004–13
Germany	*Culex pipiens s.l.*	GBMIN13858–13
South Africa	*Culex pipiens s.l.*	GMSAX808–14
South Africa	*Culex univittatus*	GBDP49678–19
South Africa	*Culex univittatus*	GBDP49679–19
Kenya	*Culex univittatus*	GBMIN57551–17
Uganda	*Culex annulioris*	GBDCU235–12
Kenya	*Culex annulioris*	GBMIN57031–17
Uganda	*Culex annulioris*	GBMIN57032–17
South Africa	*Culex poicilipes*	CULSA067–19
South Africa	*Anopheles pretoriensis*	CULSA074–19
Kenya	*Anopheles pretoriensis*	GBMIN55746–17
Senegal	*Anopheles pharoensis*	MK586014.1
Kenya	*Anopheles pharoensis*	KU380430.1
Zambia	*Anopheles pharoensis*	KR014844.1
Kenya	*Mansonia africana*	KU187126.1
Kenya	*Mansonia africana*	KU380479.1
Kenya	*Mansonia uniformis*	KU380441.1
Kenya	*Mansonia uniformis*	KU187160.1
South Africa	*Mansonia uniformis*	CULSA029–19
South Africa	*Mansonia uniformis*	CULSA035–19

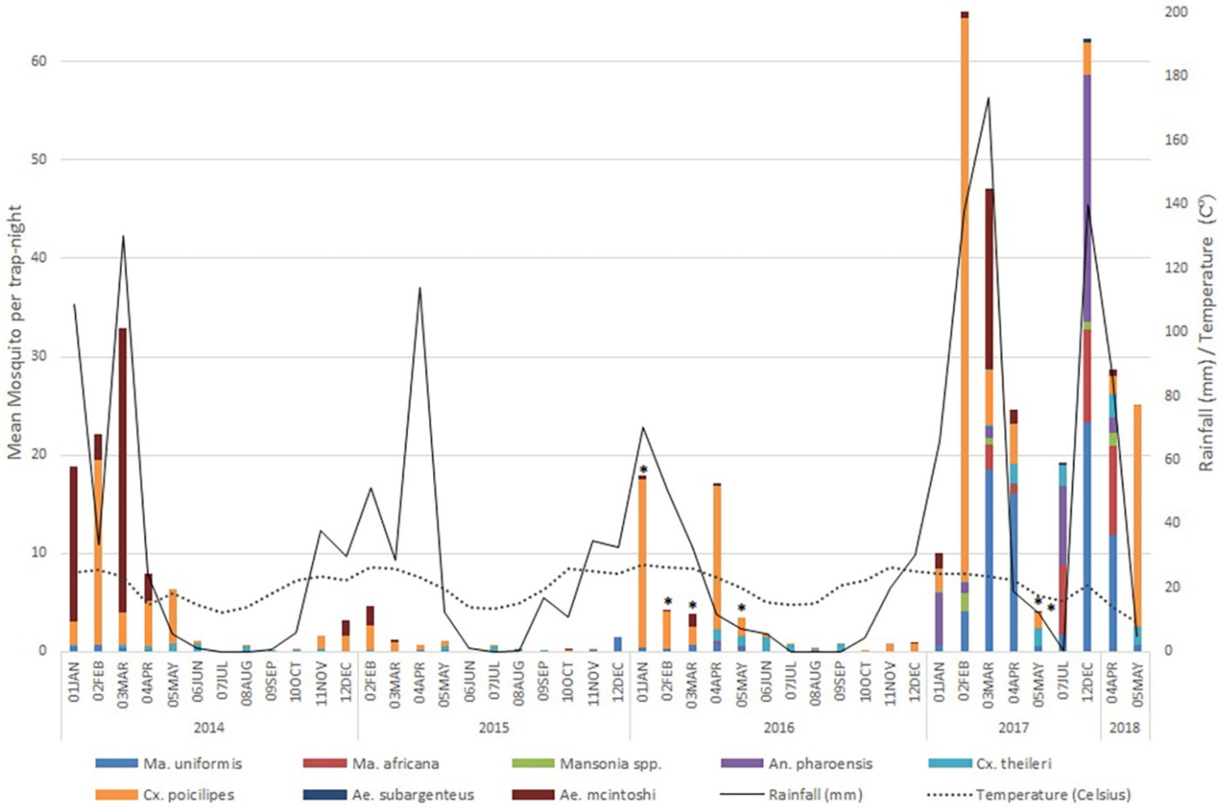

Appendix Figure 1. Mosquito abundance per trap-night, rainfall (mm) and temperature (°C) from January 2014 to May 2018, South Africa. The star represents months where were identified mosquito pools positive for Shuni virus.

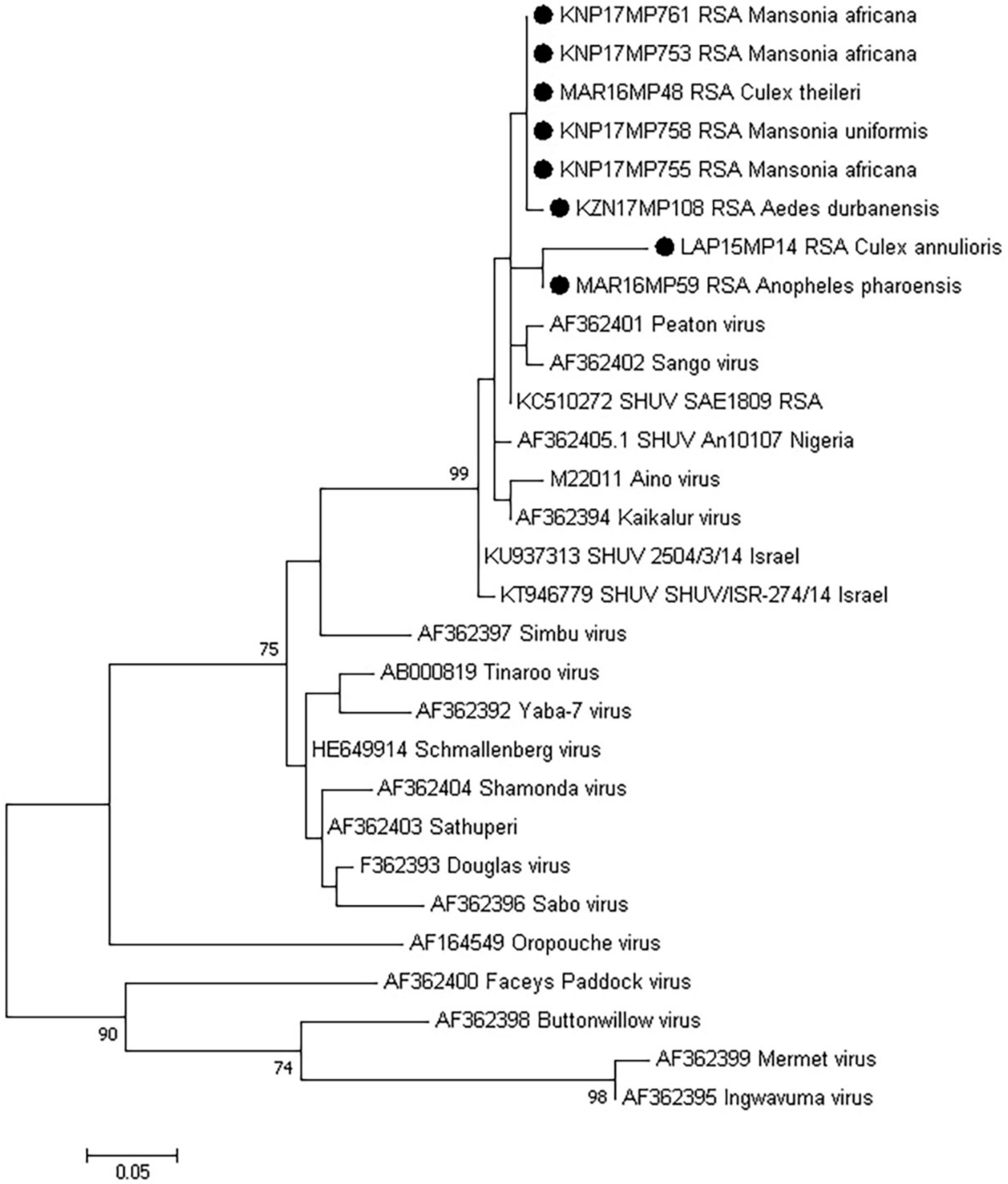

Appendix Figure 2. Phylogenetic tree of the positive mosquito homogenate pools based on the 28 sequences and 113 bp of the nucleocapsid (S) segment gene. The tree was constructed with MEGA 7, using the Maximum Likelihood method and the Kimura 2-parameter model and 1000 bootstrap replicates. The tree with the highest log likelihood (−722.25) is shown. GenBank accession numbers are indicated. Numbers on internal branches indicate bootstrap values. Samples which are part of this study are marked with a circle. The origin of SHUNV strains included are indicated and were from South Africa (RSA), Nigeria, and Israel. Other sequences were obtained from GenBank and include representative members of the Simbu serogroup.

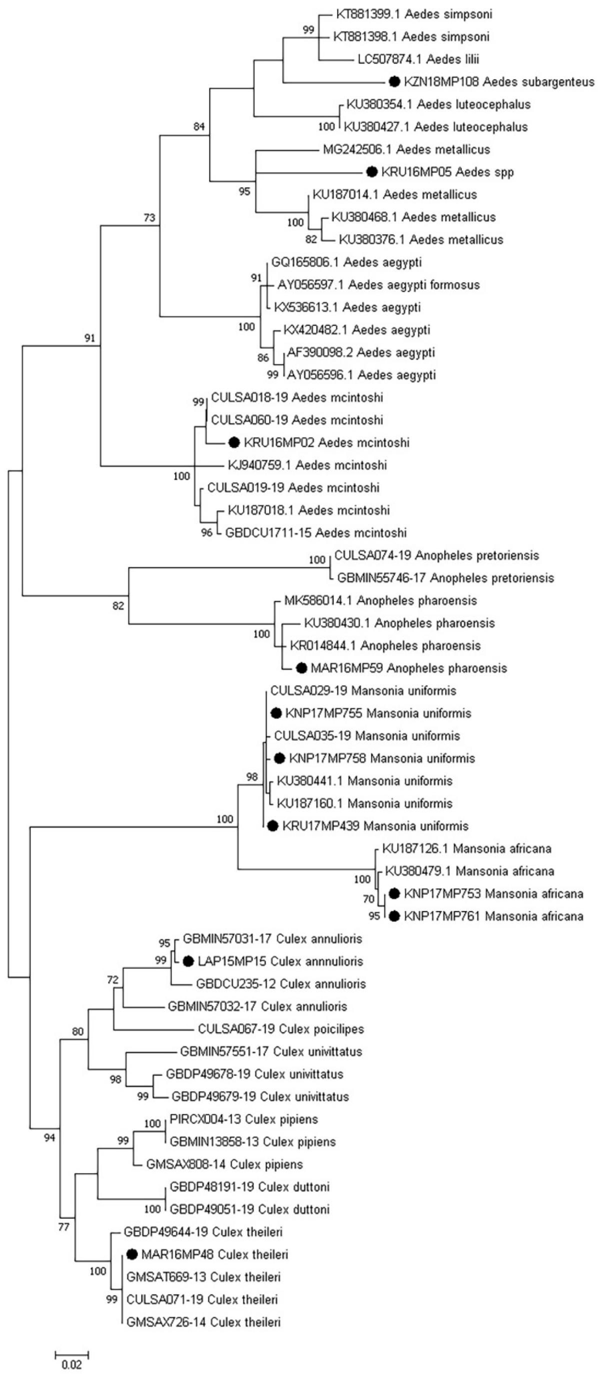

Appendix Figure 3. Phylogenetic tree of the positive mosquito homogenate pools based on 59 sequences and 517 bp of COI gene. The tree was constructed by employing the program MEGA 7, using the Maximum Likelihood method based on the General Time Reversible model and 1000 bootstrap replicates. The tree with the highest log likelihood (−3924.69) is shown. GenBank accession numbers are indicated and include representative sequences of each species identified in this study available on GenBank and/or BOLD. Numbers on internal branches indicate bootstrap values. Samples which are part of this study are marked with a circle.
